# Multi-view Subspace Clustering Analysis for Aggregating Multiple Heterogeneous Omics Data

**DOI:** 10.3389/fgene.2019.00744

**Published:** 2019-08-20

**Authors:** Qianqian Shi, Bing Hu, Tao Zeng, Chuanchao Zhang

**Affiliations:** ^1^Hubei Key Laboratory of Agricultural Bioinformatics, College of Informatics, Huazhong Agricultural University, Wuhan, China; ^2^Department of Applied Mathematics, College of Science, Zhejiang University of Technology, Hangzhou, China; ^3^Key Laboratory of Systems Biology, Institute of Biochemistry and Cell Biology, Shanghai Institute of Biological Sciences, Chinese Academy of Sciences, Shanghai, China; ^4^Shanghai Research Center for Brain Science and Brain-Inspired Intelligence, Shanghai, China; ^5^Wuhan Institute of Huawei Technologies, Wuhan, China

**Keywords:** multi-view subspace clustering analysis, data integration, heterogeneity, low-rank representation, graph diffusion

## Abstract

Integration of distinct biological data types could provide a comprehensive view of biological processes or complex diseases. The combinations of molecules responsible for different phenotypes form multiple embedded (expression) subspaces, thus identifying the intrinsic data structure is challenging by regular integration methods. In this paper, we propose a novel framework of “Multi-view Subspace Clustering Analysis (MSCA),” which could measure the local similarities of samples in the same subspace and obtain the global consensus sample patterns (structures) for multiple data types, thereby comprehensively capturing the underlying heterogeneity of samples. Applied to various synthetic datasets, MSCA performs effectively to recognize the predefined sample patterns, and is robust to data noises. Given a real biological dataset, i.e., Cancer Cell Line Encyclopedia (CCLE) data, MSCA successfully identifies cell clusters of common aberrations across cancer types. A remarkable superiority over the state-of-the-art methods, such as iClusterPlus, SNF, and ANF, has also been demonstrated in our simulation and case studies.

## Introduction

The rapid advance of high throughput technologies makes large amounts of various omics data available to study biological problems (Schuster, 2008). While, different types of data could provide complementary or common information to each other since a biological system consists of a series of highly ordered molecular and cellular events (Wang et al., 2014; Ma and Zhang, 2017; Shi et al., 2017a). Thus, compared to single data types (e.g., gene expression), the integration of multiple omics data is more likely to completely understand the molecular mechanisms underlying particular biological processes or complex diseases, and therefore offers more opportunities to better address biological or medical issues, e.g., to identify cancer subtypes with different biological or clinical outcomes (Xiong et al., 2012; Chen and Zhang, 2016; Shi et al., 2017a).

So far, quite a lot of data-integration methods have been proposed and they can be briefly summarized into two main categories: firstly, to extract signals from each data type; secondly, to acquire comprehensive information by a sample-centric integration (Arneson et al., 2017; Zhang et al., 2017a). In addition, these data integration methods mainly depend on two strategies, one is space projection method (Fan et al., 2016), and the other one is metric (similarity measures) fusion technique (Wang et al., 2014). These ideas match the nonlinear characteristics of biological systems and should really work when capturing the whole phenotype landscape.

However, their solutions to obtain the sample or gene patterns from multiple data domains are really distinct from each other. The earliest proposed methods identify multi-dimensional genomic modules (e.g., mRNA-miRNA functional pairs) (Ghazalpour et al., 2006; Kutalik et al., 2008; Li et al., 2012; Zhang et al., 2012; Chen and Zhang, 2016), which present high correlations over the samples in data sets. Such “co-modules” can only uncover common sample structures across data types and likely lead to biased clustering because much phenotype-associated differential information is missing. Later, Mo et al. developed a method, iClusterPlus (Mo et al., 2013), which considers different properties of omics data (e.g., continuous, count or binary valued variables) through corresponding linear regression models. However, some assumptions held by this method are too strong for heterogeneous tumor samples, and may also lose biologically meaningful information. As a nearly assumption-free and fast approach, SNF (Wang et al., 2014) (similarity network fusion) can overcome such issues and it uses local structure preservation method (i.e., *K*-nearest neighbors) to adjust sample similarity networks for each data type. But, SNF can only characterize pair-wise Euclidean (or other) distances in the sample neighborhoods, and is sensitive to local data noises or outliers. Recently, Ma and Zhang proposed ANF, an “update” of SNF, which incorporates weights of views for each data type (Ma and Zhang, 2017). ANF presents more general and interpretable power than SNF, but it still reserves the unstable nature of pair-wise clustering. Notably, increasing biological evidence suggests that distinct regulatory mechanisms preside over physiological phenotypes (e.g., Waddington’s canalization) or even the tumor cell states (Mark and Aviv, 2002). Cell types or patients present extremely strong heterogeneity due to the different master gene sets, implying that these individuals are scattered in multiple biological states (feature subspaces) even at a single data level (Shi et al., 2017b; Haghverdi et al., 2018). That means the pair-wise similarity measurement (e.g., in SNF) can’t capture the true heterogeneity spanning in different subspaces, further leading to inaccurate integrative clustering. Thus, the more effective integration approach is still lacking.

Motivated by above requirements from methodology and biology study, we propose a novel framework called “Multi-view Subspace Clustering Analysis (MSCA)” by using representation-based methods (e.g., low-rank representation, namely LRR) (Lin et al., 2011; Liu et al., 2013). LRR or relevant subspace clustering algorithms are originally developed and applied in image recognition (Zhang et al.; Cao et al., 2015; Gao et al., 2016; Brbić and Kopriva, 2017; Zhang et al., 2017b). These methods enable to recover the signal spaces of the images, providing a better description of the visual patterns. Furthermore, they generate a block-diagonal representation graph of samples, which measures sample similarities by linear combinations of the remaining samples, presenting more robust than pair-wise clustering. However, when applied to highly heterogenous data, such as biological omics profiles, these methods are often fragile since they assume linear embedded structures underlie the original data and can’t exploit the local geometric relationships of objects (Zhuang et al., 2015). Hence, we should improve the utility of subspace clustering to be more appropriated for biological cases. In our proposed MSCA model, we incorporate the advantage of local structure preservation to force the representations to be locally linear at each data type, and capture the integrative clustering pattern by fusing the multiple informative graphs from local sample representations. In particular, MSCA implements two steps of nonlinear pattern identification for different omics data during pattern fusion, where the multi-view is able to recover more details of systems’ complexity and heterogeneity. To validate the effectiveness of our method, we firstly applied MSCA to various synthetic datasets, and found that MSCA not only successfully recognizes the predefined subgroups with a better performance than several state-of-the-art methods, but also shows great robustness on different parameters’ variation. In addition, MSCA has demonstrated a good ability to yield biologically relevant subgroups of tumor cells of multiple origins in CCLE (Barretina et al., 2012) data set.

## Methods

### Method Overview

**Figure 1:**
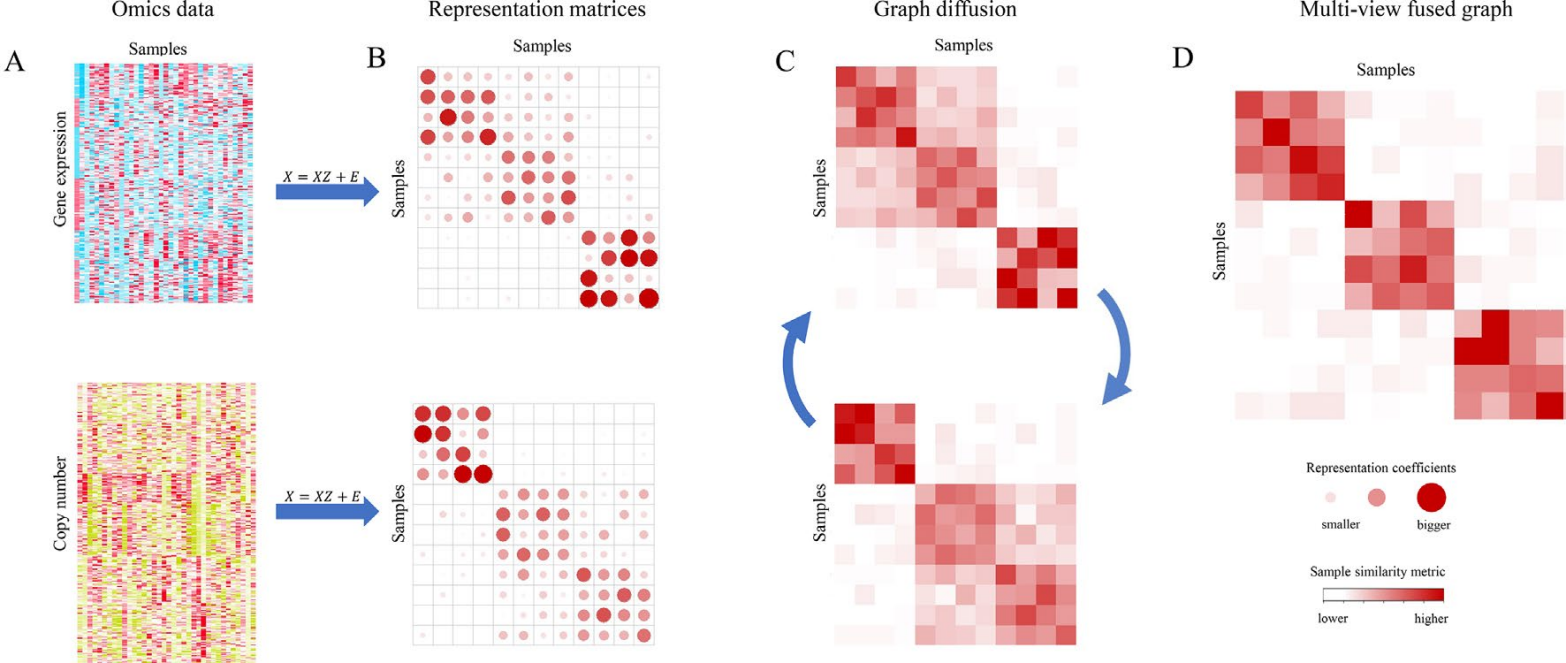
Overview of Multi-view Subspace Clustering Analysis (MSCA). **(A)** Different biological data types for the same set of samples. **(B)** Sample representation matrices for each data type. Coefficients are represented by dots, and bigger redder ones mean larger values. **(C)** Cross-graph diffusion process to integrate multiple similarity matrices, updating aggregated information iteratively. **(D)** Final integrative result when step in **(C)** reaches convergence. Color of square in graphs indicates sample-to-sample similarities. *X* denotes as each omic data matrix, and *Z* presents the representation matrix. *E* is the error matrix (see *Methods*).

MSCA takes two steps as schematically shown in **Figure 1**: i) Construction of sample representation matrix from each type of genomic profiles by a subspace clustering algorithm (**Figures 1A, B**); ii) Graph diffusion process of sample similarity matrices, which are derived from the representation matrices corresponding to all data types (**Figure 1C**). MSCA was implemented as a Matlab package and is freely available at https://github.com/ZCCQQWork/MSCA.

The representation graph *Z* of step (i) presents each single sample as a linear combination of the remaining ones in the same subspace/cluster, and therefore it can be shown as a block-diagonal and sparse matrix. Such low-rank characteristic of *Z* makes it more robust to data outliers and capable to retain more structural information of data, thus paving a good way for the next integrative. After that, MSCA implements the graph diffusion step (ii). It makes information propagate across multiple graphs in an iteration way. And this could fuse biological signals from the involved genomic data. After a few iterations, MSCA converges to the optimal graph (**Figure 1D**), as a multi-view similarity measurement, revealing the underlying relationship of samples. Note that both the steps follow nonlinear criteria, to maximize the chance of characterizing the true complexity and heterogeneity of data, and especially the common information will strengthen the supported sample patterns whereas discordant local structures will weaken their similarities.

### Extracting the Sample Representation Graph From Each Data Type

Suppose we describe a genomic profile (e.g., mRNA expression) with *h* biological measurements and *n* samples as a data matrix *X* = [*x*_1_, *x*_2_, …, *x* *_n_*], *x* *_i_* and *x* *_j_* correspond to two samples; then the representation relationships of all samples can be calculated as follows:

(1) $$\begin{array}{l} \underset{Z,E}{\min}\|Z{\|}_{*}+\lambda \|E{\|}_{2,1}\\ \text{s.t.}\{\begin{array}{c} X=\mathrm{XZ}+E\\ Z^{T}1=1\\ Z_{\mathrm{ij}}=0,(i,j)\in \bar{\Omega } \end{array} \end{array}$$

where *Z* = [*z_1_, z_2_, … ,z_n_*] is a *n × n* matrix containing all the coefficient measurements between pairs of samples *x* *_i_*(1 ≤ *i* ≤ *n*), and *z* *_i_* is a coefficient vector of sample *i*. ||**Z**||_*_ represents the nuclear norm of **Z**, i.e., the sum of all singular values of *Z*; ${\left\|E\right\|}_{2,1}=\sum_{j=1}^{n}\sqrt{\sum_{i}^{h}{\left(e_{\mathrm{ij}}\right)}^{2}}$ and is *l* *_2,1_*-norm of the error matrix *E*, where *e* *_ij_* is the (*i*,*j*)-_th_ entry of matrix *E*.

Note that, in the first constraint condition, the linear representation of samples can capture the global structure in data, thus a large similarity coefficient means the two samples are spatially close. Next in the second constraint condition, **1**, as an all-one vector, is used to normalize *Z* that ∑*_i_* *Z* *_ij_* = 1. And in the third constraint condition, $\bar{\Omega }$ denotes as the complement of Ω, where Ω is a set of edges between the samples in a predefined adjacency graph. For example, if *x* *_i_* and *x* *_j_* are not graph neighbors, we have $(i,j)\in \bar{\Omega }$. In this work, we use *K*-nearest neighbors to predetermine the sample local structure in terms of pair-wise Euclidean distances. Then, the tuning parameter *λ* is used to balance the two optimization terms, which could be selected according to their respective properties, or tuned empirically. For the selection of parameters *K* and λ, the section *Evaluation of MSCA on Synthetic Examples* has more detailed discussions. Given solving problem (1), we obtain the optimal solution Z*, which is block-diagonal indicating that samples in the same subspace are clustered together due to the comprehensive considerations/constraints of global and local data structures. The corresponding sample affinity matrix *W* is obtained by $W=\left(\left|Z^{*}\left|+\right|Z^{*T}\right|\right)/2$, which can be passed on to the next step integration.

In fact, the optimization problem (1) can be solved *via* ADMM (alternating direction method of multipliers) algorithm (Lin et al., 2010) as below. Firstly, this problem can be converted to an equivalent problem:

(2) $$\begin{array}{l} \underset{L_{{\bar{\Omega }}^{(Ζ)=0,E}}}{\min}{\left\|J\right\|}_{*}+\lambda {\left\|E\right\|}_{2,1}\\ \text{s.t.}\{\begin{array}{c} X=\mathrm{XZ}+E\\ Z^{T}1=1\\ J=Z \end{array} \end{array}$$

And its augmented Lagrangian function is:

(3) $$\begin{array}{c} L_{\mu }(Z,E,J)={\left\|J\right\|}_{*}+\lambda {\left\|E\right\|}_{2,1}+\langle Y_{1},X-\mathrm{XZ}-E\rangle +\langle Y_{2},1^{T}-1^{T}Z\rangle \\ +\langle Y_{3},Z-J\rangle +\frac{\mu }{2}\left({\left\|X-\mathrm{XZ}-E\right\|}_{F}^{2}+{\left\|1^{T}-1^{T}Z\right\|}_{F}^{2}+{\left\|Z-J\right\|}_{F}^{2}\right) \end{array}$$

where *μ* is a penalty parameter larger than 0. ||*||_F_ denotes the Frobenious norm, and *Y* *_1_*, *Y* *_2_* and *Y* *_3_* are Lagrangian multipliers corresponding to three constraints in equation (2) respectively; $L_{\bar{\Omega }}(Z)=0$ corresponds to the third constraint condition in original optimization equation (1). As known, the above problem can be minimized orderly to update the variables *Z, J, E* by fixing the other variables, respectively, according to ADMM.

Suppose at *k* times of updates, we acquire $Z^{k},J^{k},E^{k},Y_{1}^{k},Y_{2}^{k}$ and $Y_{3}^{k}$, and the alternate process with update functions can be summarized in below:

Firstly, assuming all the other five matrices are fixed, we can compute *J* *^k^* ^+1^:

(4) $$\begin{array}{c} J^{k+1}=\arg\underset{J}{\min}\|J{\|}_{*}+\langle Y_{3}^{k},Z^{k}-J\rangle +\frac{\mu^{k}}{2}\|Z^{k}-J{\|}_{F}^{2}\\ =\arg\underset{J}{\min}\|J{\|}_{*}+\frac{\mu^{k}}{2}\|Z^{k}+\frac{Y_{3}^{k}}{\mu^{k}}-J{\|}_{F}^{2} \end{array}$$

Secondly, assuming *J* *^k^* ^+1,^ Z^k,^ $Y_{1}^{k}$ are fixed, we can compute *E* *^k+1^*:

(5) $$\begin{array}{c} E^{k+1}=\arg\underset{E}{\min}\lambda \|E{\|}_{2,1}+\langle Y_{1}^{k},X-XZ^{k}-E\rangle +\frac{\mu^{k}}{2}\|X-XZ^{k}-E{\|}_{F}^{2}\\ =\arg\underset{E}{\min}\lambda \|E{\|}_{2,1}+\frac{\mu^{k}}{2}\|X-XZ^{k}+\frac{Y_{1}^{k}}{\mu^{k}}-E{\|}_{F}^{2} \end{array}$$

Thirdly, assuming *J* *^k^* ^+1,^ *E* *^k^* ^+1^, $Y_{1}^{k}$, $Y_{2}^{k}$ and $Y_{3}^{k}$ are fixed, we can compute the updated Z from following optimization problem:

(6) $$\begin{array}{c} \underset{L_{\bar{\Omega }}(Z)=0}{\min}\langle Y_{1}^{k},X-\mathrm{XZ}-E^{k+1}\rangle +\langle Y_{2}^{k},1^{T}-1^{T}Z\rangle +\langle Y_{3}^{k},Z-J^{k+1}\rangle \\ +\frac{\mu^{k}}{2}\left({\left\|X-\mathrm{XZ}-E^{k+1}\right\|}_{F}^{2}+{\left\|1^{T}-1^{T}Z\right\|}_{F}^{2}+{\left\|Z-J^{k+1}\right\|}_{F}^{2}\right) \end{array}$$

In fact, this problem is equivalent to

(7) minLΩ¯(Ζ)=0‖X−XZ−Ek+1+Y1kμk‖F2+‖1T−1TZ+Y2kμk‖F2  +‖Z−Jk+1+Y3kμk‖F2

Then, it can be further linearized with respect to *Z at Z* *^k^* based on LADMAP (linearized alternating direction method with adaptive penalty) algorithm (Lin et al., 2011):

(8) minLΩ¯(Ζ)=0〈−XT(X−XZk−Ek+1+Y1kμk)−1(1T−1TZk+Y2kμk)+(Zk−Jk+1+Y3kμk),Z−Zk〉+η2‖Z−Zk‖F2

where $\eta ={\left\|X\right\|}_{2}^{2}+{\left\|1^{T}\right\|}_{2}^{2}+1$.

In the end, we obtain *Z* *^k^* ^+1^ according to the following updating rule:

(9) Zk+1=argminLΩ¯(Z)=0<Hk,Z−Zk>+η2‖Z−Zk‖F2=argminLΩ¯(Z)=0η2‖Z−Zk+Hkη‖F2={(Zk−Hkη)ij,(i,j)∈Ω0, (i,j)∈Ω¯

where

Hk=−XT(X−XZk−Ek+1+Y1kμk)−1(1T−1TZk+Y2kμk)   +(Zk−Jk+1+Y3kμk)

Fourthly, assuming that *E* *^k^* ^+1^, *Z* *^k^* ^+1^ and *J* *^k^* ^+1^ are fixed, we can calculate simultaneously:

(10) $$Y_{1}^{k+1}=Y_{1}^{k}+\mu^{k}(X-XZ^{k+1}-E^{k+1})$$

(11) $$Y_{2}^{k+1}=Y_{2}^{k}+\mu^{k}(1^{T}-1^{T}Z^{k+1})$$

(12) $$Y_{3}^{k+1}=Y_{3}^{k}+\mu^{k}(Z^{k+1}-J^{k+1})$$

All the above subproblems can form a closed loop until convergence, and the whole step to derive the graph weight matrix *W* can be briefly summarized in Algorithm 1.

**Algorithm 1:** Algorithm to extract the sample representation matrix for each data type.

**Input:** the profile of *i*_th_ data type, i.e. $X^{i}=\left[x_{1}^{i},x_{2}^{i},...,x_{n}^{i}\right]$, tuning parameter *λ*, and nearset neighbors parameter *K*. **Output:** the sample representation matrix *W* *^i^* of *i*_th_ data type. 1. Obtain neighbors in data *X* *^i^* using *K*-nearset neighbour method, and assign the parameter Ω 2. Solve the equation (1) by updating (4), (5), (9)-(12) until the iteration converges and obtain the optimal Z* 3: Construct the sample similarity matrix *W* *^I^* by $W^{i}=\left(\left|Z^{*}\right|+\left|Z^{*T}\right|\right)/2$

### Capturing Multi-View Graph From Various Omics Data

Given *m* different genomics data types, we could obtain respective affinity matrices *W* *^i^* *, i = 1, 2, …, m* as nonlinear similarity measurements of all samples by above Algorithm 1. This step would fuse individual affinity graphs to a systematic one. The graph diffusion process is implemented like SNF ever does (Wang et al., 2014). In this step, we continue to take advantage of locality-preserving strategy and define a kernel matrix, *S*, to ensure samples in the same neighborhood still stay close across data sources. Simultaneously, we normalized the raw affinity matrix *W* to a new status matrix *P*, which keeps the original information and reduces the scale bias. Note that matrix *P* still carries the full information about the similarity of each sample to all others whereas matrix *S* only encodes the similarity to the local neighborhoods for each sample.

For the *m* different biological data types, matrices *P* *^i^* and *S* *^i^* of the *i*-th data type are obtained by equations (13) and (14) based on (*W* *^i^*, *i* = 1, 2, …, *m*).

(13) $$P^{i}(i,j)=\{\begin{array}{c} \frac{W^{i}(i,j)}{2\sum_{k\neq i}W^{i}(i,k)},j\neq i\\ 1/2,j=i \end{array}$$

(14) $$S^{i}(i,j)=\{\begin{array}{c} \frac{W^{i}(i,j)}{\sum_{k\in N_{i}}W^{i}(i,k)},j\in N_{i}\\ 0,\;\mathrm{otherwise} \end{array}$$

where *N* *_i_* is the *K* nearest neighbors of the sample *x* *_i_* based on *W* *^i^*.

The key step of MSCA is to iteratively update status matrix in graph diffusion across data types as follows:

(15) $$\begin{array}{c} P_{t+1}^{1}=S^{1}\times \left(\frac{\sum_{k\neq 1}P_{t}^{k}}{m-1}\right)\times {\left(S^{1}\right)}^{T}\\ ...\\ P_{t+1}^{i}=S^{i}\times \left(\frac{\sum_{k\neq i}P_{t}^{k}}{m-1}\right)\times {\left(S^{i}\right)}^{T}\\ ...\\ P_{t+1}^{m}=S^{m}\times \left(\frac{\sum_{k\neq m}P_{t}^{k}}{m-1}\right)\times {\left(S^{m}\right)}^{T} \end{array}$$

where $P_{t+1}^{i}$ is the status matrix of *i*-th data type after *t* + 1 iterations and $P_{1}^{i}=P^{i}$ represent the initial status matrix at *t* = 1.

The equation (15) updates the status matrices each time generating *m* parallel interchanging diffusion processes. After *t* steps, the overall status matrix or multi-view matrix *W* ^#^ is computed as:

(16) $$W^{\#}=\frac{\sum_{i=1}^{m}P_{t}^{i}}{m}$$

### Iterative Updating Process and Clustering Method

Given a series of sample representation matrices generated by Algorithm 1, the iterative integration process is summarized as Algorithm 2.

**Algorithm 2:** The Iterative Updating Process for MSCA.

**Input:** The profile of the *m* data types, i.e., $X=[X^{1},X^{2},...,X^{m}]$, tuning parameter *λ*, and nearset neighbors parameter *K*. **Output:** The multi-view similarity matrix *W* *^#^* across *m* data types 1. Computing the representation matrix *W* *^i^* (*i* = 1,2,..*m*) of each data type according to Algorithm 1 2. Updating the status matrix *P* *^i^* (*i* = 1,2...*m*) of each data type by the equation (15) until the process reaches convergence 3. Capturing the multi-view similarity matrix W^#^ by the equation (16)

Therefore, the final undirected graph *W* ^#^, involving multi-layer signals, i.e., local and global information, is capable to present the intrinsic complexity of data. The multi-view fused matrix can be applied into spectral clustering algorithm [e.g., Ratio Cuts (Ding et al., 2013)] to identify the meaningful groups of samples, e.g., prognostic different subtypes, or other potential applications.

## Results

### Evaluation of MSCA on Synthetic Examples

To demonstrate the ability of MSCA on multi-view subgroups identification, simulation experiments are conducted, with comparison to the above mentioned methods (Mo et al., 2013; Wang et al., 2014; Cao et al., 2015; Brbić and Kopriva, 2017; Ma and Zhang, 2017; Zhang et al., 2017b). In addition, the selection of parameters in MSCA has also been discussed in these synthetic examples.

#### Synthetic Data

**Figure 2:**
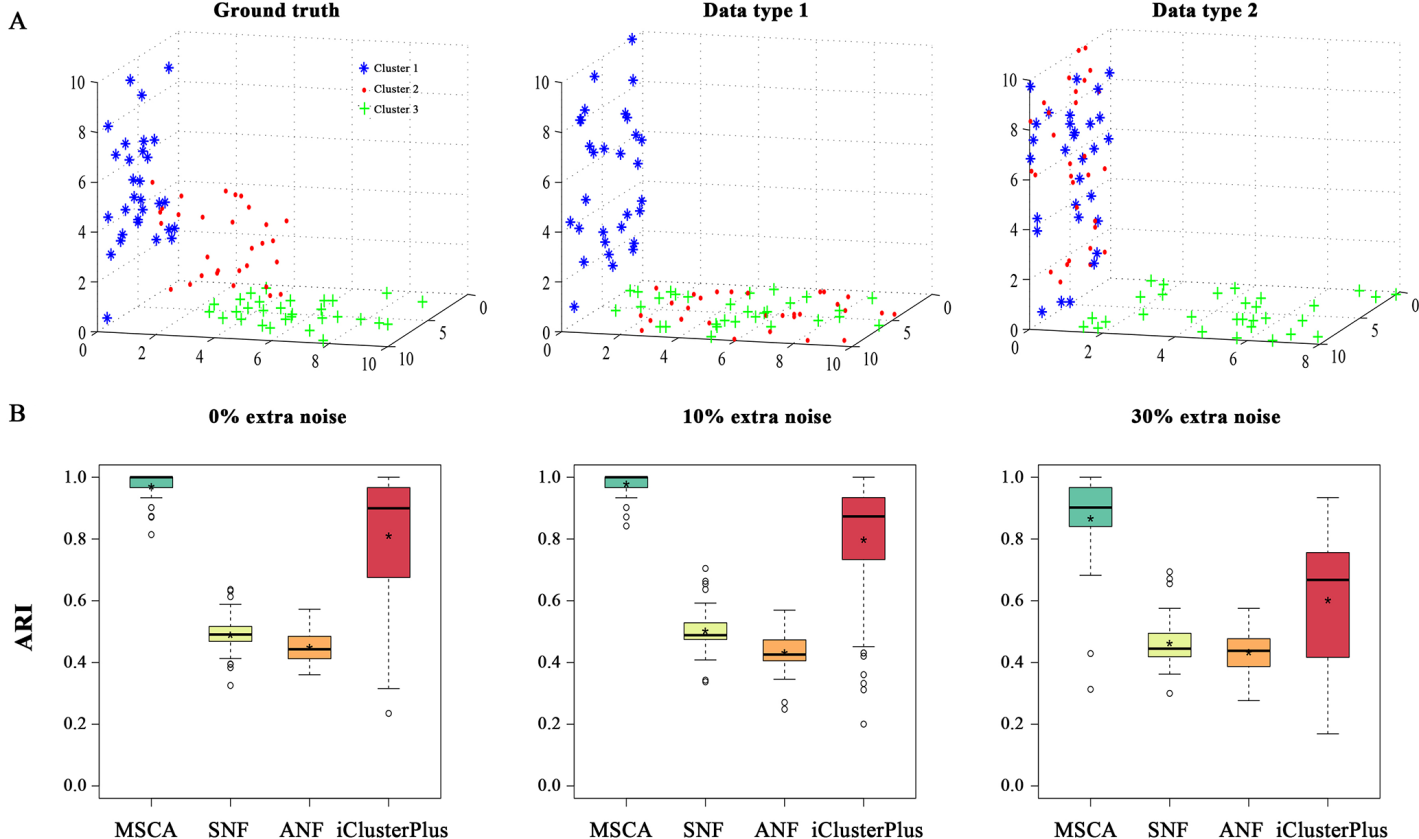
A simulation study on simData2. **(A)** 3D Illustration of sample patterns in different feature spaces. Data points, i.e., samples, are colored and shaped by their true cluster labels. Clean cluster boundaries only can be seen in an integrative affine space. Points in two clusters may be mislabeled in a single coordinated space, i.e., Cluster 2 and Cluster 3 for data type 1, Cluster 1 and Cluster 2 for type 2. **(B)** The clustering accuracy comparison among MSCA, SNF, ANF and iClusterPlus under different noise conditions, measures their effectiveness on detecting integrated sample-patterns.

Two categories of numeric data sets have been considered for a complete evaluation. Each contains two types of data and 90 samples underlying predefined sample structures by singular value decomposition (Meng et al., 2015). To preserve feature characteristics (e.g., amount, diversity and variance) of biological data types (e.g., gene expression and methylation profiles), the two data types in synthetic examples are directly generated from real data sets (i.e., GSE49278 and GSE49277) (Assié et al., 2014) (**Supplementary Information**). And each data type could provide partial but effective information to describe the whole sample patterns (e.g., type 1 and type 2 in **Figure 2A** and **Supplementary Figure S1A**). We called the “weak heterogeneity” numeric example as simData1 where samples are distributed in a single subspace and the “strong heterogeneity” one as simData2 where different manifold subspaces exist. Briefly, the 90 samples with three established clusters (namely, 1-30, 31-60, 61-90) in simData1 and simData2 are randomly selected from real data, where samples 31-90 present similar distributions from data type 1; and 1-60 appear close from data type 2. But the samples in 31-90 and 1-60 would have different embedded structures or manifold subspaces. Note that the true clusters cannot be recovered by any single data type in both synthetic examples (**Figure 2A** and **Supplementary Figure S1A**).

#### Evaluation and Comparison Based on Cluster Identification

We first applied MSCA and the other methods to the generated data sets (i.e., simData1 and simData2) with predetermined clustering structures. To avoid accidental events, both the data sets were randomly repeated 500 times under different systematic conditions (i.e., low: 0% extra noises; moderate: 10% extra noises; high: 30% extra noises), respectively. And the performance of each algorithm was measured by adjusted Rand index (ARI) (Santos and Embrechts, 2009), and a high value indicates an identical clustering. According to all the results, MSCA always succeeded to piece the information of each data type together, brilliantly distinguishing the pre-designed three clusters (**Figure 2B** and **Supplementary Figure S1B**). Given simData1 of less heterogeneity, all of the compared methods almost perform excellent (**Supplementary Figure S1B**). However, when complexity increases, a great performance difference among different methods comes out. Our MSCA model still performed accurately and robustly to identify sample patterns, even across varying noise strengths (**Figure 2B**). But the pair-wise clustering-based methods, i.e., SNF and ANF, obviously can’t recognize the multiple manifolds embedded in high-dimensional space. Even for those subspace clustering algorithms, they didn’t perform that well when integrating data sets with biological characteristics (**Supplementary Figure S2**), thus highlighting the feasibility of MSCA for biological cases. While, iClusterPlus performed the second best on accuracy, but the accuracy ranges manifested “long-tail” to expose the unstable nature of iClusterPlus. It’s probably because iClusterPlus uses random sampling procedure to solve equations (Mo et al., 2013), and is sensitive to data noises. In all, the novel nonlinear similarity measurement in MSCA is demonstrated to be robust to data noises and heterogeneity, which helps provide a more accurate multi-view for sample patterns in multi-level dataset.

#### Robustness Analysis of MSCA Under Different Parameters

**Figure 3:**
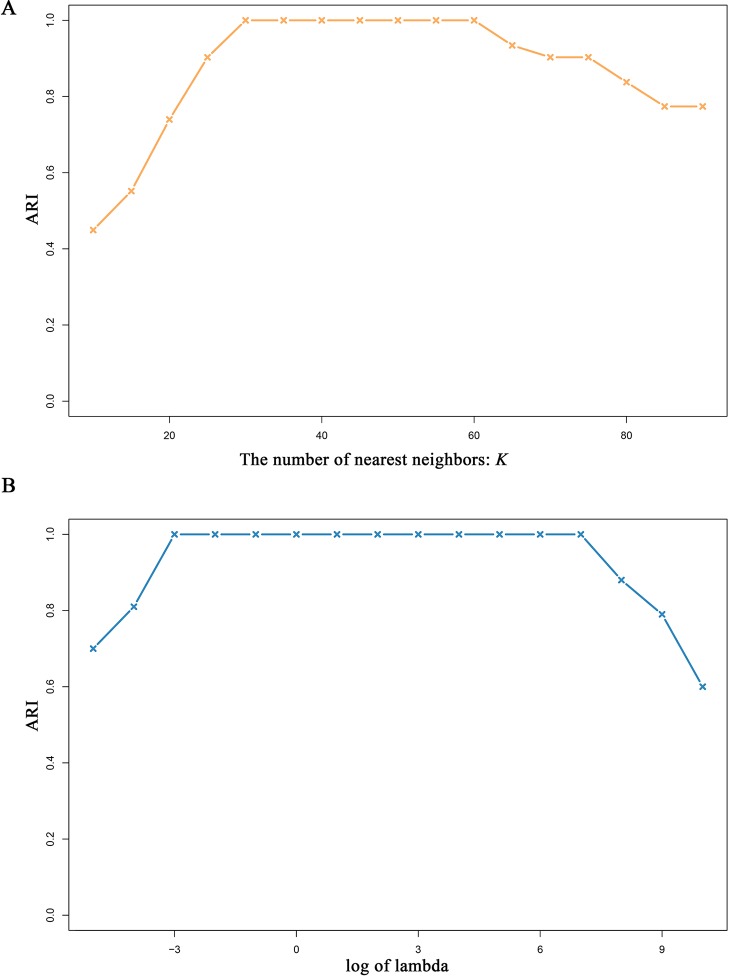
Performance of MSCA under different parameters. Varying selected nearest neighbor number *K* or the tuning parameter λ, MSCA identifies the predefined clusters in simData2.

There are two parameters, i.e., λ and *K* (see *Methods*), in MSCA model, thus it is crucially important to examine their effects on the MSCA performance. In particular, the parameter *K* determines the predefined neighborhoods, which constrains the solutions of sample representation matrices. Under different selections of *K* or λ, we use simData2 to test the robustness of MSCA. To avoid results by chance, we repeated 1,000 times and take the average ARI values as evaluation measurement. According to all the results (**Figure 3**), MSCA performs stable and accurate in a wide range of *K* and λ. Once again, the advantage of combining low-rank presentation and local preservation makes MSCA more parameter-independent, and brings a novel light on developing new bioinformatic tools for integrating heterogeneous biological data.

### Study on CCLE Data

To demonstrate the effectiveness of MSCA to address practical issues, we have applied MSCA to CCLE datasets (Barretina et al., 2012) with the matched mRNA expression profiles by Affymetrix Human Genome U133 Plus 2.0 array and copy number data by Affymetrix SNP Array 6.0. Though it contains thousands of cell lines, we only kept 415 cell lines, whereby more than 25 cells have the same tissues of origin (**Supplementary Table S1**). For each tissue, we obtained its specific expressed genes from two databases: The Human Protein Atlas (Uhlen et al., 2015) and PaGenBase (Pan et al., 2013). Several organs belong to upper aerodigestive tract cancer (UADT), including tongue, trachea and esophagus etc., thus, all their gene sets were treated as UADT specific genes. While tumor associated genes were collected from GeneCards (Safran et al., 2010) and top 100 by the provided relevance scores were selected to illustrate corresponding aberration patterns among different subgroups. We adopted one-sided Wilcoxon signed-rank test to identify the tissue-specific genes between one of the clusters and all the remaining ones. More highly expressed genes with *P* < 0.05 (adjusted by FDR) indicate the cluster strongly correlated with a certain tissue of origin. Similarly, differential expression or copy number was calculated using two-sided Wilcoxon signed-rank test for each single gene. A significant *P*-value shows gene expression or copy number in one group dominates the other cell lines and we regard those differential genes with *P* < 0.05 (after FDR correction) as cluster markable features. Though clusters may share markable features, we count the number of shared clusters to measure the inter-cluster heterogeneity.

**Figure 4:**
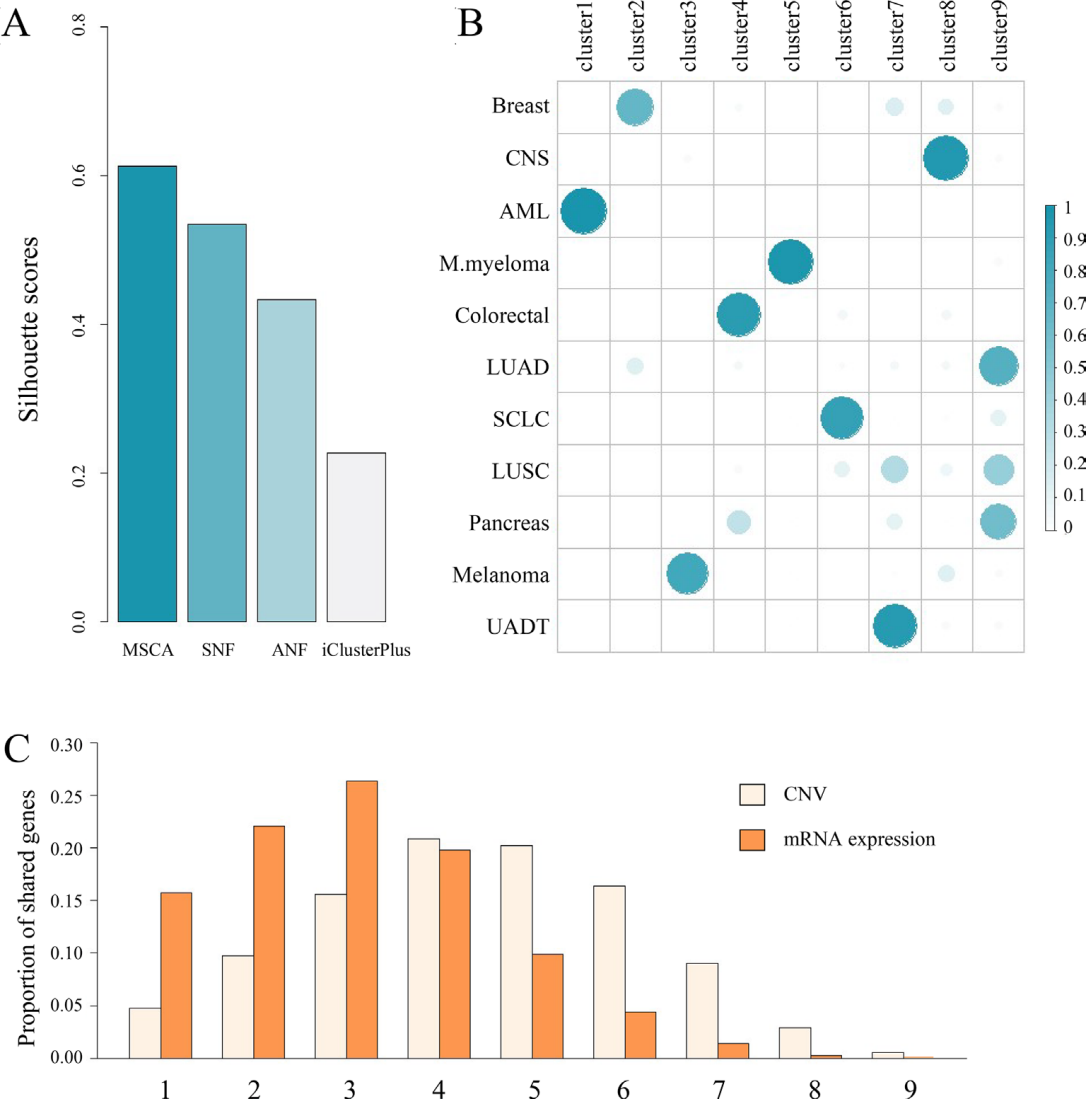
A case study on CCLE dataset. **(A)** Comparison of the integrative clustering results obtained by MSCA, SNF, ANF and iClusterPlus. Note that the number of clusters is the same, i.e., 9 for the four methods. **(B)** Illustration of associations between histological origins (i.e., rows) and integrated cell clusters (i.e., columns). Sum of each row equals 1. **(C)** A brief summary of remarkable features across clusters. *x*-axis indicates the number of shared groups. Breast, breast cancer; CNS, central nervous systems; AML, acute myelocytic leukemia; M.myeloma, multiple myeloma; Colorectal, colorectal cancer; LUAD, lung adenocarcinoma; SCLC, small cell lung cancer; LUSC, lung squamous cell carcinoma; Pancreas, pancreas cancer; UADT, upper aerodigestive tract cancer.

Firstly, we used the silhouette score (Rousseeuw, 1999) to evaluate how coherent the identified clusters are, and then we assigned the cell lines into nine clusters (**Supplementary Figure S3**). Among the compared methods, we observed MSCA had a better silhouette score, indicating superior subgroup identification for CCLE samples (**Figure 4A**). Then, we compared the integrative clusters with the original tissue groups (**Figure 4B**), and found some cell lines still manifest high lineage dependency (Pearson correlation 0.42). For example, all the AML or M. myeloma cell lines are assigned to single clusters (i.e., cluster1 and cluster5, respectively), separating from other solid tumor ones. Accordingly, the cluster1 preserves about 77% blood genes and cluster5 holds 85% lymph associated genes (**Supplementary Figure S4**). Besides, the characteristic preservation of tissue specificity for some clusters can explain their homogeneity in turn. But beyond all that, we can see different histological cancer cell lines are grouped into the same integrative clusters because they share gene alterations (**Supplementary Figures S5, S6**). Notably, the markable features between clusters, especially those copy number variants (**Figure 4C**), tend to be held by only few clusters, revealing strong heterogeneity between MSCA identified clusters (*P*-value < 10^−12^ and < 10^−23^ for expression and copy number data respectively, identified by sample shifting test for 5,000 times). Thus, the integrated pan-cancer analysis by MSCA may challenge the tissue original separation and indicate the common molecular aberrations across tumor types.

## Discussion

It’s widely acceptable that integration of distinct types of biological data could provide more complete information to understand system complexity and disease heterogeneity (Ghazalpour et al., 2006; Kutalik et al., 2008; Li et al., 2012; Zhang et al., 2012; Zhang et al., 2017c). Over the past decades, the integration methods have progressed to get closer to biological details, from focusing on common information to specific signals, from critical hypothesis to assumption-free, and from linear models to nonlinear methods, etc. However, it is still a challenging task for bioinformatics to more accurately capture the underlying sample/gene structures from multiple omics data.

Here, we propose the MSCA model with the capacity to identify precise manifolds of samples in data space. In fact, our MSCA method is very similar to a previously published method, SNF (and ANF), which attempts to recognize sample patterns based on cross-view diffusion. However, the biggest difference is that SNF regards all the samples in the same feature space, nevertheless MSCA considers therein embedded multiple subspaces, i.e., different functional molecule sets. We carried out both synthetic examples and a real cancer dataset to demonstrate the capacities of MSCA. In the *in silico* studies, MSCA effectively fused the concordant information associated in certain sample subgroups and outperformed several state-of-the-art integrative methods, in terms of clustering accuracy and robustness. In real case study, the sample patterns derived by MSCA correspond to biological differences using independent knowledge and analytic methods. Beyond that, we believe it can also help other studies which need integration of various data sources, in addition to complex diseases.

Though MSCA implements two nonlinear steps, proven to be effective in theory and practice, the problem of over-learning might still exist because we use the local similarities twice (see *Methods*). Such design may lead to bias when data types contain a lot of shared noises, which is worth careful consideration and improvement. Furthermore, MSCA has currently dealt with continuous data types (e.g., mRNA expression, copy number variant), the effectiveness on other forms of data, e.g., binary data (somatic mutation), category data (clinical covariates), still needs to be continuously improved.

## Author Contributions

CZ and QS completed the majority of the project and wrote the article. TZ and BH revised the article.

## Funding

This paper was supported by the National Natural Science Foundation of China (No. 61802141), Natural Science Foundation of Hubei Province (No. 2018CFB098) and Huazhong Agricultural University Scientific & Technological Self-innovation Foundation (No. 2662017QD043).

## Conflict of Interest Statement

The authors declare that the research was conducted in the absence of any commercial or financial relationships that could be construed as a potential conflict of interest.
