# Supplementary material for: Multi-view Subspace Clustering Analysis for Aggregating Multiple Heterogeneous Omics Data
Source: Front Genet. 2019 Aug 20;10:744. doi: 10.3389/fgene.2019.00744 (PMC6712585; doi:10.3389/fgene.2019.00744)
Supplement: Supplementary file 1 [file Table_1.pdf]

Multi-view subspace clustering analysis for aggregating multiple heterogeneous omics data

Qianqian Shi[1*], Bing Hu[2], Tao Zeng[3], Chuanchao Zhang[4*]

[1]Hubei Key Laboratory of Agricultural Bioinformatics, College of Informatics, Huazhong Agricultural University, Wuhan 430070, China
[2]Department of Applied Mathematics, College of Science, Zhejiang University of Technology, Hangzhou 310023, China
[3]Key Laboratory of Systems Biology, Institute of Biochemistry and Cell Biology, Shanghai Institute of Biological Sciences, Chinese Academy of Sciences, Shanghai 200031, China
[4]Wuhan Institute of Huawei Technologies, Wuhan 430070, China
[*]Corresponding authors:
Qianqian Shi: qqshi@mail.hzau.edu.cn;
Chuanchao Zhang: chaozhangchuan@163.com

S1. Synthetic examples

Simulation datasets were generated as in [1]. Firstly, two real biological datasets of different platforms, e.g., gene expression and methylation profiles, were prepared including GSE49278 and GSE49277 [2]. 90 samples were randomly selected and 2 types of data were constructed, referred to as X_1 and X_2, where rows present biological measurements and columns for samples. Then, singular value decomposition (SVD: $X = UDV$) was applied in data matrices X_1, and X_2, respectively.

$$X_1 = U_1 D_1 V_1 \quad \text{and} \quad X_2 = U_2 D_2 V_2$$

In order to preserve the true biological characteristics in data, we kept the matrices Us and modified the matrices Vs with 3 pre-defined clusters, i.e., samples 1-30 for cluster 1, 31-60 for cluster 2, and 61-90 for cluster 3. Cluster 2 and cluster 3 can't be distinguished in data type 1, while cluster 1 and cluster 2 appear more close from data type 2. Only the combination of two data types can recover the full cluster structures.

In order to better mimic different types of heterogeneity (i.e., embedded subspaces), we generated two types of simulation data sets, wherein the weak

heterogeneous example denoted as simData1 with samples in a single subspace and the strong one as simData2 underlying three manifold subspaces. Such difference is implemented by modifying the matrices Vs when generating two data sets.

For simData1, the modification of Vs is as follows:

$$v_{ij} = mean^k + value_{ij} \tag{1}$$

where $value_{ij} \sim N(0,1)$ represents random biases for expression of element i in sample j; $mean^k \in \{2,6\}$ represents the average expression level in cluster k (k=1, 2) for each data type. For example, samples 1-30 belong to same cluster and 31-90 are assigned into the other cluster in data type 1; and in data type 2, samples 1-60 are grouped together and 61-90 as the other cluster. Base on equation (1), the pre-designed matrices V_{sim1} and V_{sim2} represent corresponding sample structures in 2 types of data for simData1.

For simData2, we need to construct three different subspaces in data of strong heterogeneity. For convenience, we selected three rows of matrices Vs, which have the largest singular values, as the defined subspaces, and the remaining values are all equal to 0. Then, the pattern matrices V_{sim1} and V_{sim2} could be generated as follows:

$$V_{sim1} = \begin{bmatrix} 10*\text{rand}(1,30)+5, 10*\text{rand}(1,30)+5,\ 10*\text{rand}(1,30)+5 \\ 0*\text{rand}(1,30), 10*\text{rand}(1,30)+5,\ 10*\text{rand}(1,30)+5 \\ 10*\text{rand}(1,30)+5, 0*\text{rand}(1,30),\ 0*\text{rand}(1,30) \\ \mathbf{0} \end{bmatrix}$$

$$V_{sim2} = \begin{bmatrix} 10*\text{rand}(1,30)+5, 10*\text{rand}(1,30)+5,\ 10*\text{rand}(1,30)+5 \\ 0*\text{rand}(1,30), 0*\text{rand}(1,30),\ 10*\text{rand}(1,30)+5 \\ 10*\text{rand}(1,30)+5, 10*\text{rand}(1,30)+5,\ 0*\text{rand}(1,30) \\ \mathbf{0} \end{bmatrix}$$

where the function rand(n,m) return a n-by-m matrix of pseudorandom uniform values.

Finally, the simulated data sets X_{layer1} and X_{layer2} for simData1 and simiData2 were thus created by:

$$X_{layer1} = U_1 D_1 V_{sim1} \quad \text{and} \quad X_{layer2} = U_2 D_2 V_{sim2}$$

Table S1. Description of CCLE data used in this study.

Tumor type	Sample size
breast carcinoma	33
central nervous system (glioma grade IV)	35
acute myeloid leukaemia	34
multiple myeloma	29
colorectal adenocarcinoma	43
lung adenocarcinoma	47
lung small cell carcinoma	53
lung squamous cell carcinoma	27
pancreas ductal carcinoma	26
melanoma	60
upper aerodigestive tract squamous cell carcinoma	28

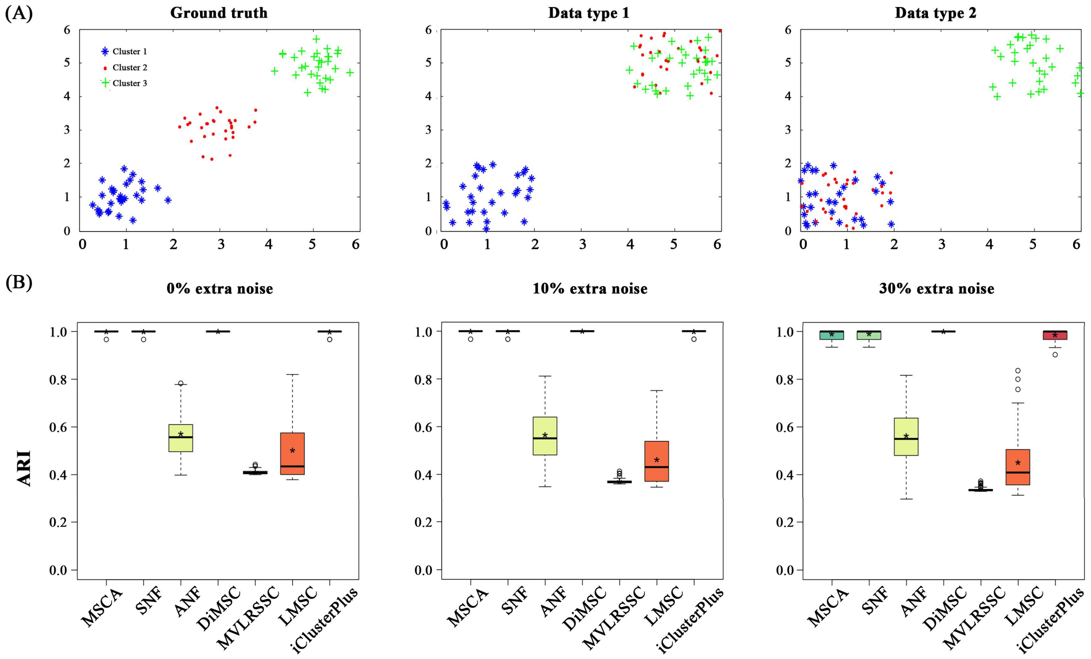

Figure S1. A simulation study on simData1. (A) 2D Illustration of sample patterns in different feature spaces. Data points, i.e., samples, are colored and shaped by their true cluster labels. Clean cluster boundaries only can be seen in a integrative affine space. Points in two clusters may be mislabeling in a single coordinated space, i.e., Cluster 2 and 3 for data type 1, Cluster 1 and cluster 2 for type 2. (B) The clustering accuracy comparison among MSCA, SNF, ANF, iClusterPlus and other multi-view subspace clustering algorithms under different noise conditions, measures their effectiveness on detecting integrated sample-patterns.

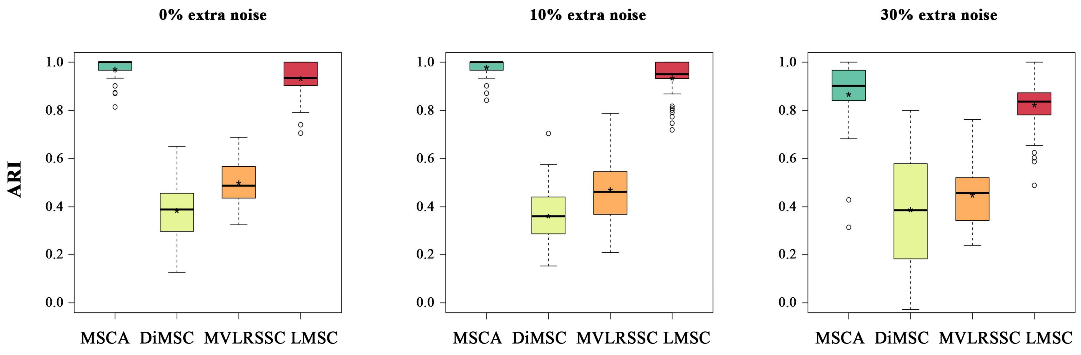

Figure S2. Comparison of several multi-view subspace clustering algorithms (i.e., DiMSC, MVLRSSC and LMSC) on simData2.

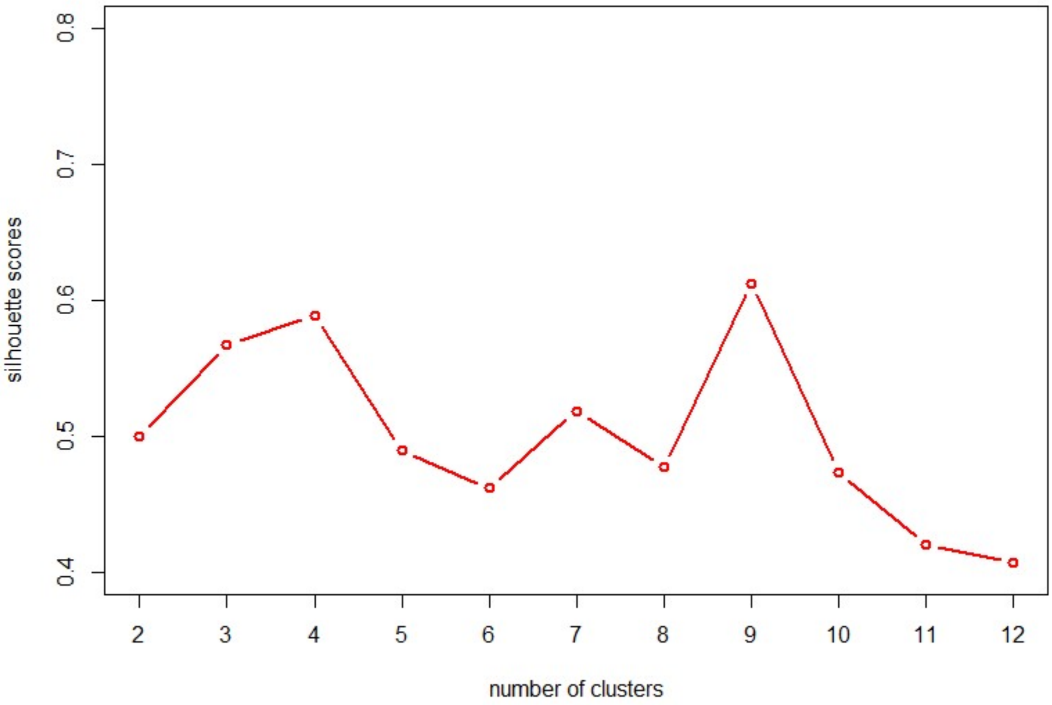

Figure S3. Determination of number of clusters for CCLE data.

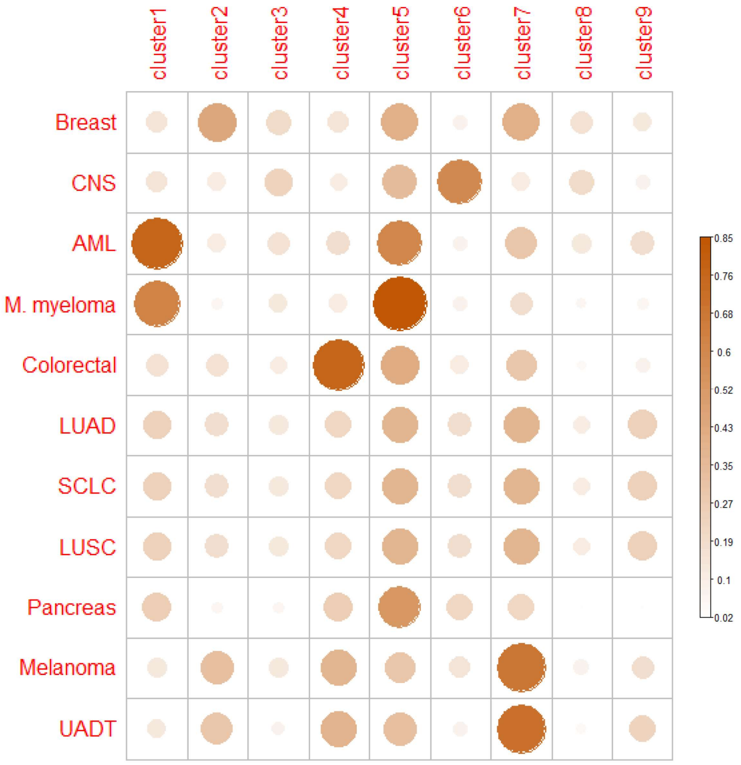

Figure S4. The proportion of cluster over-expressed gene sets to tissue-specific gene sets. A high value indicates strong lineage-dependency.

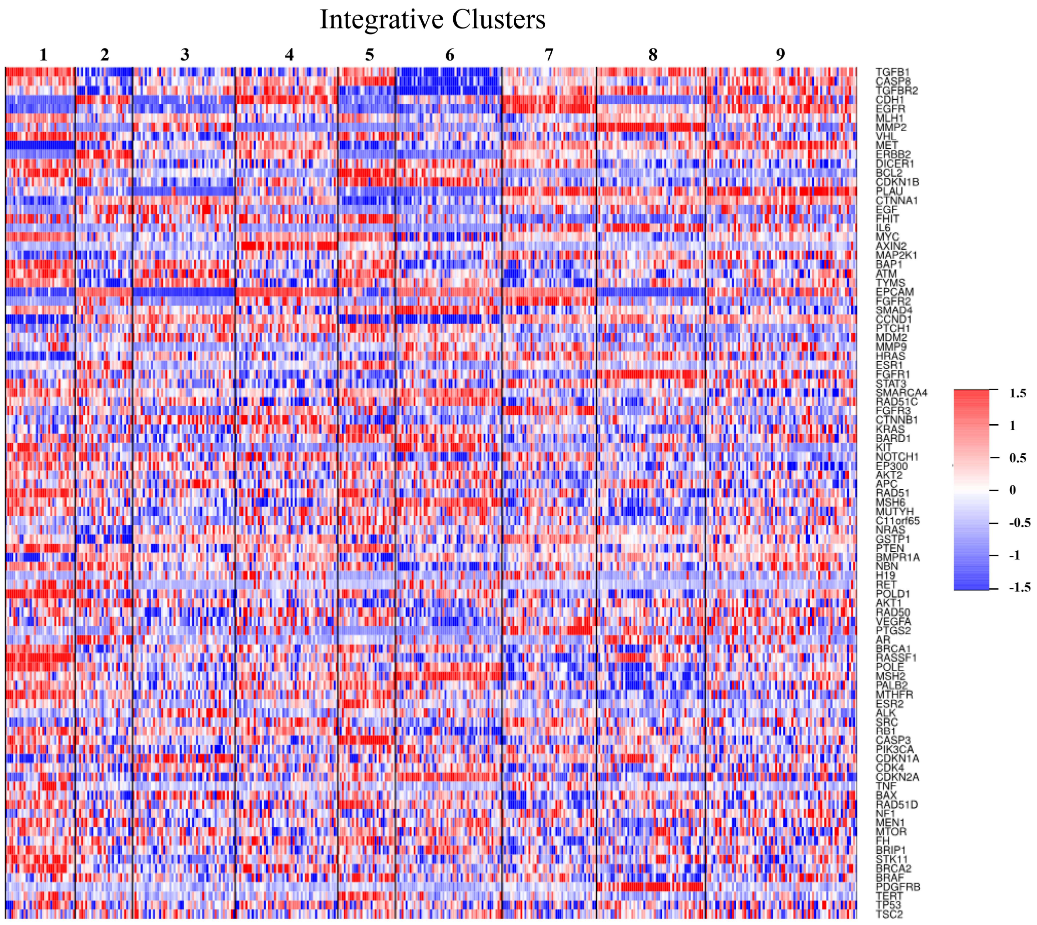

Figure S5. The expression patterns of top 100 cancer related genes. Rows are genes and columns are cell line samples sorted by cluster assignment**.**

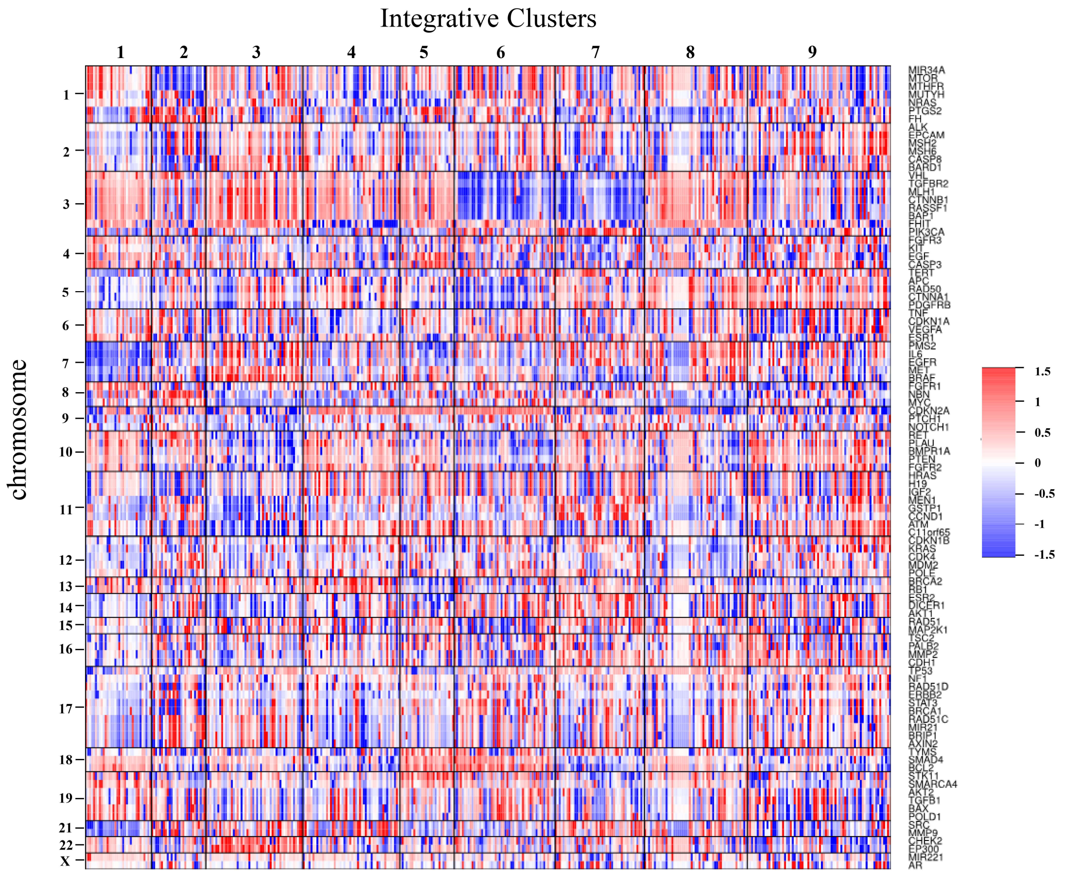

Figure S6. The CNV patterns of top 100 cancer related genes. Rows are genes and columns are cell line samples sorted by cluster assignment**.**

Reference

1. Meng, C., et al., *moCluster: Identifying Joint Patterns Across Multiple Omics Data Sets.* J Proteome Res, 2016. **15**(3): p. 755-65.
2. Guillaume, A., et al., *Integrated genomic characterization of adrenocortical carcinoma.* Nature Genetics, 2014. **46**(6): p. 607-612.
